# Supplementary material for: Modelling local and general quantum mechanical properties with attention-based pooling
Source: Commun Chem. 2023 Nov 29;6:262. doi: 10.1038/s42004-023-01045-7 (PMC10686994; doi:10.1038/s42004-023-01045-7)
Supplement: Supplementary file 2 — Supplementary Information [file 42004_2023_1045_MOESM2_ESM.pdf]

Modelling local and general quantum mechanical properties with attention-based pooling – Supplementary Information

SI 1 Train and validation metrics

The train and validation metrics (MAE) for the models presented in Table 1, including sum pooling and ABP, are reported in Supplementary Table 1 for SchNet, and in Supplementary Table 2 for DimeNet++.

Supplementary Table 1: Train and Validation MAE (mean ± standard deviation from 5 data random splits) for QM7b, QM8, QM9, QMugs, and MD17 (best MAEs in bold) using SchNet. Abbreviations: maximal absorption, MA; self-consistent screening, SCS; atomization, atom.; excitation, exc.; ionization, ion.; units, u.; malondialdehyde, MDA.

**QM7b**	**SchNet Train**		**SchNet Validation**	
Task – level of theory (unit)	**Sum**	**ABP**	**Sum**	**ABP**
Atom. energy – ZINDO/s (meV)	4.014 ± 0.783	3.338 ± 0.265	2.522 ± 0.345	2.193 ± 0.263
Electron affinity – ZINDO (meV)	1.043 ± 0.321	0.501 ± 0.101	1.829 ± 0.147	1.167 ± 0.112
Exc. energy at MA – ZINDO (meV)	28.388 ± 4.313	14.986 ± 9.454	38.083 ± 1.580	31.209 ± 1.593
First exc. energy – ZINDO (meV)	1.077 ± 0.225	0.619 ± 0.070	2.240 ± 0.090	1.321 ± 0.033
HOMO – GW (meV)	1.277 ± 0.567	0.773 ± 0.106	3.185 ± 0.214	2.369 ± 0.059
HOMO – PBE0 (meV)	0.877 ± 0.210	0.601 ± 0.059	2.811 ± 0.176	2.118 ± 0.082
HOMO – ZINDO/s (meV)	0.904 ± 0.255	0.587 ± 0.100	2.581 ± 0.133	1.715 ± 0.074
Ion. potential – ZINDO/s (meV)	1.400 ± 0.416	0.667 ± 0.104	3.774 ± 0.121	2.497 ± 0.107
LUMO – GW (meV)	1.036 ± 0.418	0.601 ± 0.189	2.895 ± 0.278	2.099 ± 0.105
LUMO – PBE0 (meV)	0.653 ± 0.105	0.482 ± 0.087	1.944 ± 0.107	1.446 ± 0.076
LUMO – ZINDO/s (meV)	0.880 ± 0.124	0.513 ± 0.086	1.456 ± 0.123	0.877 ± 0.071
MA intensity – ZINDO (arbitrary u.)	0.035 ± 0.006	0.012 ± 0.014	0.057 ± 0.003	0.049 ± 0.002
Polarizability – DFT/PBE0	0.025 ± 0.003	0.025 ± 0.007	0.036 ± 0.001	0.028 ± 0.002
Polarizability – SCS	0.027 ± 0.007	0.023 ± 0.005	0.025 ± 0.003	0.020 ± 0.001
**QM8**	**Sum**	**ABP**	**Sum**	**ABP**
E1-CAM (meV)	1.255 ± 0.260	0.816 ± 0.110	3.171 ± 0.303	2.205 ± 0.071
E1-CC2 (meV)	1.422 ± 0.348	0.899 ± 0.067	3.645 ± 0.181	2.658 ± 0.174
E1-PBE0/def2SVP (meV)	1.466 ± 0.573	1.055 ± 0.224	3.589 ± 0.327	2.583 ± 0.238
E1-PBE0/def2TZVP (meV)	1.610 ± 0.528	1.073 ± 0.158	3.642 ± 0.332	2.524 ± 0.130
E2-CAM (meV)	1.579 ± 0.281	1.170 ± 0.192	4.343 ± 0.186	3.136 ± 0.131
E2-CC2 (meV)	2.660 ± 0.588	1.341 ± 0.336	5.608 ± 0.281	4.164 ± 0.260
E2-PBE0/def2SVP (meV)	2.084 ± 0.629	1.392 ± 0.358	4.797 ± 0.258	3.394 ± 0.194
E2-PBE0/def2TZVP (meV)	1.580 ± 0.353	1.170 ± 0.200	4.488 ± 0.187	3.327 ± 0.131
f1-CAM (meV)	3.083 ± 0.837	1.941 ± 0.631	9.597 ± 0.632	7.568 ± 0.799
f1-CC2 (meV)	2.997 ± 0.587	2.775 ± 1.605	10.645 ± 0.621	8.639 ± 0.960
f1-PBE0/def2SVP (meV)	3.940 ± 1.767	2.280 ± 0.791	9.429 ± 0.860	7.534 ± 0.489
f1-PBE0/def2TZVP (meV)	3.578 ± 1.702	1.658 ± 0.429	9.088 ± 0.674	7.387 ± 0.751
f2-CAM (meV)	5.990 ± 1.494	3.169 ± 1.345	22.372 ± 1.217	17.051 ± 0.892
f2-CC2 (meV)	13.903 ± 4.208	4.264 ± 1.682	28.667 ± 1.158	22.748 ± 0.733
f2-PBE0/def2SVP (meV)	6.106 ± 1.056	3.648 ± 1.543	20.733 ± 0.439	16.033 ± 0.680
f2-PBE0/def2TZVP (meV)	6.138 ± 2.943	4.019 ± 1.274	21.099 ± 0.845	16.697 ± 0.546
**QM9**	**Sum**	**ABP**	**Sum**	**ABP**
HOMO – DFT (meV)	11.005 ± 1.119	9.903 ± 0.945	35.997 ± 0.893	29.355 ± 0.513
LUMO – DFT (meV)	13.837 ± 0.561	12.830 ± 1.487	32.644 ± 0.597	26.747 ± 0.831
**QMugs**	**Sum**	**ABP**	**Sum**	**ABP**
HOMO – DFT (meV)	27.776 ± 3.950	27.562 ± 0.586	64.057 ± 1.326	51.388 ± 0.608
LUMO – DFT (meV)	28.937 ± 0.527	22.572 ± 0.540	61.774 ± 0.347	46.825 ± 0.650
Total Energy – DFT (E_h)	11.702 ± 3.609	4.029 ± 1.186	3.995 ± 1.259	1.787 ± 0.721
**MD17 (energies)**	**Sum**	**ABP**	**Sum**	**ABP**
Aspirin – CCSD (kcal/mol)	2.392 ± 0.302	1.742 ± 0.148	3.942 ± 0.008	3.619 ± 0.073
Benzene – CCSD(T) (kcal/mol)	0.410 ± 0.085	0.315 ± 0.047	0.327 ± 0.050	0.325 ± 0.030
Ethanol – CCSD(T) (kcal/mol)	0.386 ± 0.062	0.395 ± 0.099	0.682 ± 0.037	0.675 ± 0.030
MDA – CCSD(T) (kcal/mol)	0.425 ± 0.086	0.545 ± 0.055	0.874 ± 0.013	0.878 ± 0.029
Toluene – CCSD(T) (kcal/mol)	1.020 ± 0.218	0.684 ± 0.109	0.974 ± 0.135	0.917 ± 0.057

Supplementary Table 2: Train and Validation MAE (mean ± standard deviation from 5 data random splits) for QM7b, QM8, QM9, QMugs, and MD17 (best MAEs in bold) using DimeNet++. Abbreviations: maximal absorption, MA; self-consistent screening, SCS; atomization, atom.; excitation, exc.; ionization, ion.; units, u.; malondialdehyde, MDA.

**QM7b**	**DimeNet++ Train**		**DimeNet++ Validation**	
Task – level of theory (unit)	**Sum**	**ABP**	**Sum**	**ABP**
Atom. energy – ZINDO/s (meV)	3.061 ± 1.056	4.509 ± 0.663	3.108 ± 0.319	1.925 ± 0.446
Electron affinity – ZINDO (meV)	0.587 ± 0.096	0.503 ± 0.098	1.209 ± 0.027	1.128 ± 0.052
Exc. energy at MA – ZINDO (meV)	15.879 ± 6.390	15.069 ± 3.295	31.045 ± 1.578	29.824 ± 2.234
First exc. energy – ZINDO (meV)	0.614 ± 0.159	0.704 ± 0.160	1.389 ± 0.080	1.321 ± 0.052
HOMO – GW (meV)	0.773 ± 0.110	0.729 ± 0.053	2.409 ± 0.086	2.241 ± 0.039
HOMO – PBE0 (meV)	0.700 ± 0.062	0.588 ± 0.096	2.100 ± 0.054	1.960 ± 0.039
HOMO – ZINDO/s (meV)	0.584 ± 0.197	0.588 ± 0.100	1.669 ± 0.066	1.653 ± 0.058
Ion. potential – ZINDO/s (meV)	0.908 ± 0.127	0.837 ± 0.163	2.705 ± 0.091	2.471 ± 0.055
LUMO – GW (meV)	0.782 ± 0.255	0.644 ± 0.113	2.084 ± 0.017	2.131 ± 0.070
LUMO – PBE0 (meV)	0.546 ± 0.179	0.453 ± 0.071	1.472 ± 0.082	1.430 ± 0.105
LUMO – ZINDO/s (meV)	0.407 ± 0.069	0.474 ± 0.072	0.877 ± 0.040	0.859 ± 0.046
MA intensity – ZINDO (arbitrary u.)	0.028 ± 0.008	0.031 ± 0.005	0.048 ± 0.002	0.050 ± 0.002
Polarizability – DFT/PBE0	0.024 ± 0.005	0.029 ± 0.004	0.035 ± 0.003	0.028 ± 0.003
Polarizability – SCS	0.020 ± 0.004	0.028 ± 0.005	0.029 ± 0.002	0.025 ± 0.002
**QM8**	**Sum**	**ABP**	**Sum**	**ABP**
E1-CAM (meV)	0.808 ± 0.078	0.665 ± 0.055	1.971 ± 0.099	1.869 ± 0.090
E1-CC2 (meV)	0.872 ± 0.099	0.833 ± 0.131	2.337 ± 0.147	2.285 ± 0.072
E1-PBE0/def2SVP (meV)	0.910 ± 0.100	0.775 ± 0.100	2.234 ± 0.090	2.183 ± 0.045
E1-PBE0/def2TZVP (meV)	0.856 ± 0.154	0.738 ± 0.133	2.204 ± 0.026	2.141 ± 0.044
E2-CAM (meV)	1.123 ± 0.249	0.955 ± 0.176	2.887 ± 0.116	2.921 ± 0.086
E2-CC2 (meV)	1.378 ± 0.317	0.974 ± 0.109	3.919 ± 0.115	3.911 ± 0.142
E2-PBE0/def2SVP (meV)	1.201 ± 0.260	0.984 ± 0.262	3.153 ± 0.107	3.118 ± 0.059
E2-PBE0/def2TZVP (meV)	1.152 ± 0.154	0.905 ± 0.044	3.068 ± 0.047	3.090 ± 0.039
f1-CAM (meV)	1.525 ± 0.445	1.943 ± 0.523	6.279 ± 0.663	6.439 ± 0.751
f1-CC2 (meV)	2.234 ± 0.296	2.090 ± 0.246	7.390 ± 0.393	7.510 ± 0.469
f1-PBE0/def2SVP (meV)	1.390 ± 0.484	1.527 ± 0.317	6.343 ± 0.425	6.246 ± 0.448
f1-PBE0/def2TZVP (meV)	1.515 ± 0.482	1.608 ± 0.489	6.318 ± 0.589	6.295 ± 0.371
f2-CAM (meV)	3.508 ± 1.177	3.195 ± 0.889	15.267 ± 0.712	15.390 ± 0.372
f2-CC2 (meV)	3.507 ± 0.951	5.384 ± 2.139	19.957 ± 0.711	20.725 ± 0.604
f2-PBE0/def2SVP (meV)	3.255 ± 1.093	3.812 ± 1.036	14.007 ± 0.537	14.565 ± 0.655
f2-PBE0/def2TZVP (meV)	3.631 ± 1.317	3.712 ± 0.986	14.830 ± 0.575	14.782 ± 0.621
**QM9**	**Sum**	**ABP**	**Sum**	**ABP**
HOMO – DFT (meV)	6.993 ± 0.127	6.054 ± 0.500	23.613 ± 0.320	21.712 ± 0.501
LUMO – DFT (meV)	7.667 ± 0.662	8.312 ± 1.141	20.384 ± 0.155	20.011 ± 0.466
**QMugs**	**Sum**	**ABP**	**Sum**	**ABP**
HOMO – DFT (meV)	12.361 ± 0.495	11.158 ± 1.170	24.158 ± 0.257	22.859 ± 0.758
LUMO – DFT (meV)	11.027 ± 0.800	11.152 ± 0.656	21.207 ± 0.464	20.567 ± 0.326
Total Energy – DFT (E_h)	3.523 ± 1.176	4.367 ± 2.327	1.283 ± 0.649	1.805 ± 1.214
**MD17 (energies)**	**Sum**	**ABP**	**Sum**	**ABP**
Aspirin – CCSD (kcal/mol)	0.611 ± 0.148	0.592 ± 0.277	2.849 ± 0.019	2.473 ± 0.133
Benzene – CCSD(T) (kcal/mol)	0.205 ± 0.022	0.166 ± 0.026	0.263 ± 0.006	0.262 ± 0.041
Ethanol – CCSD(T) (kcal/mol)	0.205 ± 0.022	0.204 ± 0.033	0.558 ± 0.005	0.567 ± 0.018
MDA – CCSD(T) (kcal/mol)	0.305 ± 0.067	0.325 ± 0.062	1.025 ± 0.008	1.087 ± 0.044
Toluene – CCSD(T) (kcal/mol)	0.419 ± 0.054	0.414 ± 0.082	0.979 ± 0.012	0.933 ± 0.029

SI 2 Resource utilisation and scaling

We report the training time per epoch and the consumed memory for SchNet and DimeNet++ models on the QM7b and QM8 datasets in Supplementary Figure 1.

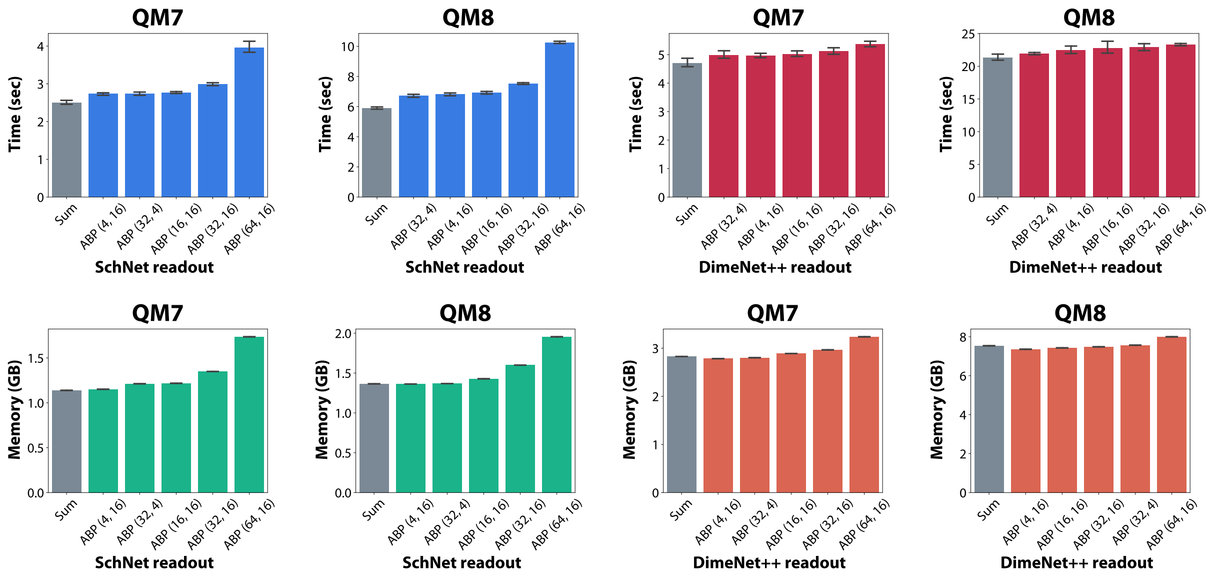

Supplementary Figure 1: Time spent training per epoch (seconds) and memory consumption (GB) of SchNet and DimeNet++ models with standard (sum) and ABP readouts for QM7b and QM8. Several configurations of ABP (hidden dimension per attention head, number of attention heads) are included for comparison, and all ABP readouts use 2 self attention blocks (SABs). Results are reported for 10 different runs of the same model configuration. All models use a batch size of 128. All the models are trained on a single GPU. Error bars represent a 95% confidence interval.

SI 3 Mean pooling metrics

The test set metrics (MAE) for mean pooling (with sum pooling results replicated from Table 1, are reported in Supplementary Table 3. For the QMugs models, we used an early stopping threshold (also called patience) of 10 epochs due to the large resource utilisation and slow training. It is possible that the test set performance could improve further with a more generous early stopping setting – however, this is unrealistic due to the large computational cost of training models such as DimeNet++ on QMugs and is unlikely to lead to large differences.

Supplementary Table 3: Test MAE (mean ± standard deviation from 5 data random splits) for QM7b, QM8, QM9, QMugs, and MD17 (best MAEs in bold) for sum and mean pooling. Abbreviations: maximal absorption, MA; self-consistent screening, SCS; atomization, atom.; excitation, exc.; ionization, ion.; units, u.; malondialdehyde, MDA.

**QM7b**	**SchNet**		**DimeNet++**	
Task (level of theory)	**Sum**	**Mean**	**Sum**	**Mean**
Atom. energy – ZINDO/s (meV)	3.859 ± 1.526	11.690 ± 3.328	4.268 ± 0.806	4.382 ± 1.113
Electron affinity – ZINDO (meV)	2.045 ± 0.240	2.215 ± 0.405	1.358 ± 0.074	1.332 ± 0.177
Exc. energy at MA – ZINDO (meV)	40.196 ± 2.018	33.438 ± 1.232	32.359 ± 0.681	32.401 ± 1.266
First exc. energy – ZINDO (meV)	2.429 ± 0.124	2.604 ± 0.601	1.571 ± 0.130	1.596 ± 0.105
HOMO – GW (meV)	3.311 ± 0.190	2.868 ± 0.094	2.475 ± 0.085	2.607 ± 0.473
HOMO – PBE0 (meV)	3.039 ± 0.131	2.669 ± 0.203	2.224 ± 0.056	2.241 ± 0.129
HOMO – ZINDO/s (meV)	2.639 ± 0.191	2.431 ± 0.229	1.849 ± 0.236	1.841 ± 0.102
Ion. potential – ZINDO/s (meV)	4.145 ± 0.326	3.781 ± 0.261	3.131 ± 0.206	3.080 ± 0.210
LUMO – GW (meV)	3.198 ± 0.313	2.471 ± 0.197	2.267 ± 0.179	2.220 ± 0.134
LUMO – PBE0 (meV)	2.042 ± 0.153	1.843 ± 0.184	1.610 ± 0.136	1.494 ± 0.155
LUMO – ZINDO/s (meV)	1.783 ± 0.164	1.931 ± 0.260	1.022 ± 0.083	1.144 ± 0.115
MA intensity – ZINDO (arbitrary u.)	0.062 ± 0.004	0.055 ± 0.004	0.050 ± 0.003	0.052 ± 0.002
Polarizability – DFT/PBE0 ($Å^3$)	0.055 ± 0.013	0.080 ± 0.029	0.041 ± 0.007	0.039 ± 0.006
Polarizability – SCS ($Å^3$)	0.040 ± 0.010	0.065 ± 0.024	0.038 ± 0.007	0.036 ± 0.007
**QM8**	**Sum**	**Mean**	**Sum**	**Mean**
E1-CAM (meV)	2.528 ± 0.105	2.506 ± 0.096	2.017 ± 0.137	2.015 ± 0.105
E1-CC2 (meV)	2.961 ± 0.089	3.062 ± 0.111	2.356 ± 0.087	2.498 ± 0.221
E1-PBE0/def2SVP (meV)	2.744 ± 0.098	2.917 ± 0.071	2.213 ± 0.092	2.297 ± 0.125
E1-PBE0/def2TZVP (meV)	2.811 ± 0.122	2.810 ± 0.088	2.198 ± 0.060	2.331 ± 0.175
E2-CAM (meV)	3.675 ± 0.066	3.858 ± 0.106	2.976 ± 0.129	3.105 ± 0.116
E2-CC2 (meV)	4.775 ± 0.339	4.871 ± 0.160	3.866 ± 0.153	4.129 ± 0.138
E2-PBE0/def2SVP (meV)	3.992 ± 0.176	4.027 ± 0.219	3.275 ± 0.110	3.353 ± 0.167
E2-PBE0/def2TZVP (meV)	3.873 ± 0.121	3.999 ± 0.206	3.173 ± 0.067	3.157 ± 0.037
f1-CAM (meV)	8.887 ± 0.747	8.445 ± 0.373	6.782 ± 0.378	6.916 ± 0.498
f1-CC2 (meV)	10.116 ± 0.237	9.462 ± 0.364	7.787 ± 0.258	7.833 ± 0.479
f1-PBE0/def2SVP (meV)	8.500 ± 0.585	8.045 ± 0.597	6.612 ± 0.560	6.848 ± 0.437
f1-PBE0/def2TZVP (meV)	8.375 ± 0.542	7.890 ± 0.568	6.919 ± 0.284	6.806 ± 0.420
f2-CAM (meV)	21.171 ± 0.813	20.078 ± 0.646	16.108 ± 0.799	17.056 ± 0.539
f2-CC2 (meV)	25.029 ± 0.825	25.039 ± 0.685	20.698 ± 1.039	21.529 ± 0.615
f2-PBE0/def2SVP (meV)	19.163 ± 1.141	18.569 ± 0.697	14.930 ± 0.762	15.362 ± 0.962
f2-PBE0/def2TZVP (meV)	19.679 ± 0.490	19.048 ± 0.660	15.455 ± 0.709	16.095 ± 0.755
**QM9**	**Sum**	**Mean**	**Sum**	**Mean**
HOMO – DFT (meV)	35.985 ± 1.071	32.788 ± 1.163	23.711 ± 0.303	23.187 ± 0.606
LUMO – DFT (meV)	33.505 ± 0.885	28.743 ± 1.097	20.832 ± 0.667	20.661 ± 1.547
**QMugs**	**Sum**	**Mean**	**Sum**	**Mean**
HOMO – DFT (meV)	65.094 ± 1.243	55.368 ± 0.309	24.536 ± 0.260	25.530 ± 0.943
LUMO – DFT (meV)	62.022 ± 0.830	53.026 ± 1.101	21.491 ± 0.557	23.419 ± 0.925
Total Energy – DFT (E_h)	9.051 ± 2.848	3.935 ± 0.653	3.352 ± 1.764	2.081 ± 1.132
**MD17 (energies)**	**Sum**	**Mean**	**Sum**	**Mean**
Aspirin – CCSD (kcal/mol)	3.935 ± 0.128	3.955 ± 2.610	2.537 ± 0.061	2.760 ± 0.144
Benzene – CCSD(T) (kcal/mol)	0.486 ± 0.295	0.808 ± 0.159	0.290 ± 0.034	0.315 ± 0.056
Ethanol – CCSD(T) (kcal/mol)	0.676 ± 0.035	0.929 ± 0.034	0.576 ± 0.026	0.603 ± 0.069
MDA – CCSD(T) (kcal/mol)	0.937 ± 0.082	1.010 ± 0.214	1.058 ± 0.026	1.125 ± 0.123
Toluene – CCSD(T) (kcal/mol)	1.529 ± 0.494	1.355 ± 0.088	1.132 ± 0.046	1.239 ± 0.077
