# Supplementary material for: Modelling local and general quantum mechanical properties with attention-based pooling
Source: Commun Chem. 2023 Nov 29;6:262. doi: 10.1038/s42004-023-01045-7 (PMC10686994; doi:10.1038/s42004-023-01045-7)
Supplement: Supplementary file 3 — Description of Additional Supplementary Files [file 42004_2023_1045_MOESM3_ESM.pdf]

Description of Additional Supplementary Files

File name: Supplementary Data 1

Description: Numerical source data for Figure 2.

File name: Supplementary Data 2

Description: Numerical source data for Figure 3 and Supplementary Figure 1.
